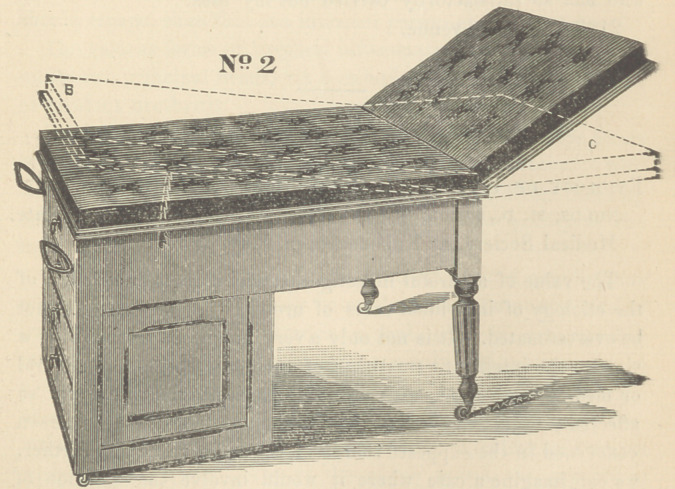# A New Operating Table

**Published:** 1883-07

**Authors:** Franklin H. Martin

**Affiliations:** Clinical Lecturer in the Gynæcological Department of South Side Dispensary, Chicago; 2139 Wabash Avenue


2139 Wabash Ave.

---

### ARTICLE V.

A NEW OPERATING TABLE. By FRANKLIN H. MARTIN, M.D., Clinical Lecturer in the Gynæcological Department of South Side Dispensary, Chicago.

The accompanying wood cuts give a fair representation of a combined office bed and gynæcological and general operating table that I have for some time used in my office. In presenting this table to the profession, I make no particular claim for originality, except in the table as a whole. It is the successful combination of numerous valuable ideas into one compact piece of office furniture that particularly recommends it.

The cuts explain themselves. No. 2 represents the combination as a bed. The extension (c), by a suitable ratchet combination, can be lowered or raised to any angle with the bed of the table; when extended on a level with the bed, it makes an excellant general operating table; when not in use, it can be lowered to right angle to plane of bed, leaving an excellent gynæcological table, as represented in No. 1. It has the ordi-

No. 1

dary convenient proportions. The bed is nicely and firmly upholstered and covered with rep or leather. The lower drawer

No. 2

(D) can be used as a step in mounting the table; the foot supporters, or stirrups, are made at such an angle that by them the knees of the patient are naturally widely separated. The foot supports are removed easily, when not in use.

For Sims' position the table possesses superior advantages. The front end of the bed of the table can be raised and retained at any angle by the side ratchet bar (see dotted lines in cut 1, B), thereby elevating the hips of the patient. At the same time the buttocks can be still more elevated by the arrangement represented by the dotted lines in cut 1 (A). By this simple arrangement the patient is retained in so desirable a position that I have many times been able, without an assistant, to make a satisfactory examination with Sims' speculum.

The table, as here illustrated, makes a very nice piece of office furniture. It is artistically manufactured of black walnut, highly polished, with nickel-plated trimmings, contains a suit of useful drawers for instruments and chemicals, is nicely upholstered, and in every way would be an ornament to any office. I may be pardoned if I say that it does great credit to the manufacturer, Mr. Wm. Proctor, of the firm of Proctor & Wood, of this city, who has so satisfactorily carried out my idea.

2139 Wabash Avenue.

## Figures and Tables

**No. 1 f1:**
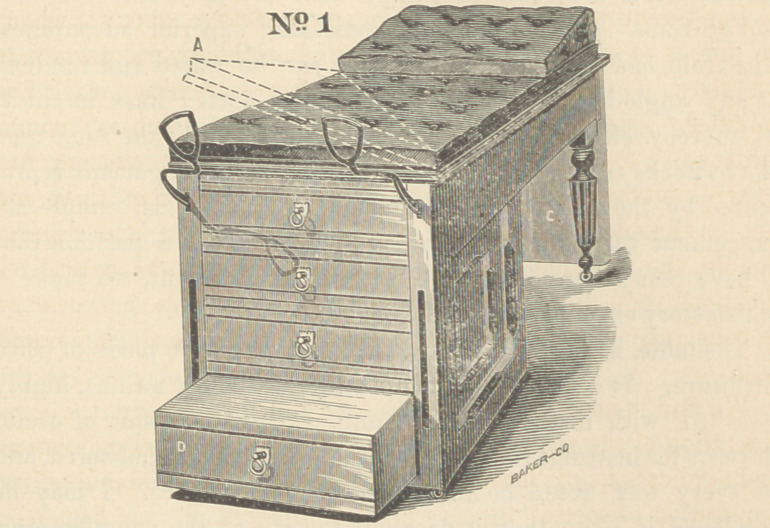


**No. 2 f2:**